# BIRC5 drives cell-cycle dysregulation and represents a novel molecular target in retinoblastoma

**DOI:** 10.3389/fonc.2026.1854143

**Published:** 2026-05-21

**Authors:** Yingtong Chen, Xiaohan Li, Fuhua Zhong, Xiaoya Wang, Qiang Wang, Di Cao, Shaowei Dong, Chengyue Zhang, Huibin Song, Chen Lin

**Affiliations:** 1 The Second Clinical Medical College of Jinan University, Department of Ophthalmology, Shenzhen People’s Hospital, Shenzhen, Guangdong, China; 2 Department of Pathology, Shengjing Hospital, China Medical University, Shenyang, China; 3 Department of Thoracic Surgery, Shenzhen People’s Hospital (The First Affiliated Hospital, Southern University of Science and Technology; The Second Clinical Medical College, Jinan University), Shenzhen, Guangdong, China; 4 Department of Ophthalmology, Beijing Children’s Hospital, Capital Medical University, National Center for Children’s Health, Beijing, China; 5 Department of Pediatric Research, Shenzhen Children’s Hospital, Shenzhen, Guangdong, China; 6 Department of Ophthalmology, Shenzhen People’s Hospital (The First Affiliated Hospital, Southern University of Science and Technology; The Second Clinical Medical College, Jinan University), Shenzhen, Guangdong, China

**Keywords:** BIRC5 (Survivin), cell cycle dysregulation, molecular targeted therapy, retinoblastoma, single-cell RNA sequencing

## Abstract

**Aims and objectives:**

Retinoblastoma is the most common pediatric intraocular malignancy, although it remains a rare disease overall, yet the molecular targets and therapeutic vulnerabilities sustaining its most aggressive proliferative cell states remain incompletely defined. We aimed to identify actionable molecular regulators and potential therapeutic targets of malignant retinoblastoma progression using integrated single-cell transcriptomics and functional validation.

**Methods:**

We analyzed single-cell RNA sequencing data from 189,431 cells derived from 13 retinoblastoma tumors and 3 normal fetal retina samples. Data integration was performed using Harmony, followed by clustering, differential expression, and functional enrichment analyses. BIRC5 function was evaluated through overexpression and siRNA-mediated knockdown in retinoblastoma cell lines (Y79, WERI-Rb1), with assessment of proliferation, migration, invasion, and cell cycle–related proteins.

**Results:**

Clustering identified 10 retinal cell types, including cone precursor-like, cone-like, and two MKI67^+^ proliferative RB subpopulations enrichment of G2/M-phase genes and chromosomal instability pathways. MKI67^+^ cells2 displayed hyperactivated cell cycle genes, chromosomal instability, and mitotic checkpoint dysregulation. BIRC5 (survivin) was selectively overexpressed in 93.7% of MKI67^+^ cells2 (mean expression 1.733 ± 0.643), while minimally expressed in other retinal cells. Functional assays demonstrated that BIRC5 overexpression promoted proliferation, invasion, migration, and CCND1/CDK4 activation, while suppressing CHEK2–p21–mediated checkpoint control. Conversely, BIRC5 knockdown induced G1 cell cycle arrest and increased apoptotic activity, accompanied by activation of checkpoint pathways.

**Conclusion:**

This study defines BIRC5 as a cell-state–specific regulator of malignant proliferation in retinoblastoma and provides a mechanistic rationale for targeting survivin in highly proliferative tumor subpopulations.

## Introduction

Retinoblastoma (RB) is the most common pediatric intraocular malignancy, although it is a rare disease overall, with an incidence of approximately 1 in 15,000 to 20,000 live births worldwide (1). This dual characterization reflects the fact that, while retinoblastoma is the most frequent intraocular malignancy in children, it remains uncommon at the population level. It is initiated by biallelic inactivation of the RB1 tumor suppressor gene, a critical regulator of G1/S transition and terminal differentiation in retinal progenitor cells (2). Although RB1 loss is a prerequisite for tumor initiation, it does not fully account for the observed heterogeneity in tumor growth kinetics, lineage identity, and response to therapy (3). This variability suggests the presence of cooperating genetic or epigenetic alterations that promote malignant progression beyond the initial RB1 inactivation. Historically considered genetically simple, RB tumors were thought to harbor few somatic mutations. However, recent bulk and single-cell transcriptomic studies have revealed substantial intra-tumoral heterogeneity, cell cycle diversity, and lineage plasticity, particularly among proliferating tumor cells (4, 5). Notably, scRNA-seq studies have identified transcriptionally distinct RB subpopulations with high expression of proliferation-associated genes such as MKI67, TOP2A, and CDK1, alongside variable activation of DNA damage and mitotic checkpoint pathways (6, 7). Moreover, chromatin accessibility assays have uncovered differential enhancer usage linked to cone photoreceptor lineage programs (RXRG, ARR3, OPN1MW), suggesting a developmentally constrained cell of origin (8). Despite this, the cone precursor model fails to explain the emergence of MKI67^+^, CCNB1^+^, and BIRC5^+^ tumor subpopulations with markedly elevated proliferative indices, chromosomal instability, and attenuated checkpoint control. These clusters often exhibit suppressed expression of CHEK2, GADD45A, and CDKN1A genes that mediate DNA damage response and G1/S arrest, indicating selective evasion of cell cycle surveillance (9). Additionally, copy number variation (CNV) profiling at the single-cell level has revealed subclonal gains on chromosome 6p (encompassing DEK, E2F3) and losses on 16q, which correlate with aggressive clinical behavior and early metastatic potential. A major unresolved question is how proliferative RB subpopulations, particularly the MKI67 high clusters, are transcriptionally maintained and hierarchically organized (10). Specifically, the functional roles of upregulated regulators such as BIRC5, HMGB2, UBE2C, and CDCA3 remain uncharacterized in RB, despite their known roles in mi-totic fidelity and resistance to apoptosis in other embryonal tumors. To address these gaps, we performed an integrated single-cell transcriptomic analysis of 13 retinoblas-toma tumors derived from three independent public datasets (GSE249995, n = 4; GSE168434, n = 7; PRJNA737188, n = 2) together with three normal fetal retina samples (E-MTAB-7316), yielding a combined dataset of 189,431 high-quality single cells. Through unsupervised clustering, gene module scoring, and pseudotime trajectory analysis, we identified a discrete MKI67^+^ cells2 subpopulation uniquely present in RB tumors. This cluster is marked by coordinated overexpression of BIRC5, AURKB, TPX2, and KIF2C, a transcriptional signature consistent with chromosomal passenger com-plex activity and defective mitotic exit. We further performed functional perturbation assays in RB cell lines to test the necessity of BIRC5 for maintaining malignant proliferation. The present study defines lineage-restricted transcriptional regulators that sustain high-risk RB subtypes and nominate therapeutically actionable dependencies grounded in single-cell lineage architecture.

## Methods

Single-cell RNA sequencing data used in this study were obtained from publicly available datasets containing 13 retinoblastoma tumors and three normal fetal retina samples. In addition, three retinoblastoma tissues and paired adjacent tissues collected at Shenzhen People’s Hospital were used exclusively for immunofluorescence validation experiments, rather than for single-cell sequencing analysis. The process has been approved by the ethics committee of Shenzhen People’s Hospital (2025-671-01), and all relevant patients have signed informed consent forms.

### Single-cell transcriptomic data collection and preprocessing

To build a comprehensive transcriptomic atlas of retinoblastoma and normal fetal retina, we aggregated publicly available single-cell RNA-seq datasets from both tumor and control groups. The datasets included PRJNA737188 (7), GSE168434 (5), GSE249995 (10), and EMTAB7316 (11), all generated using the 10x Genomics Chromium platform for single-cell RNA sequencing. Raw FASTQ files were processed with Cell Ranger (v9.0.1) to generate formatted gene expression matrices. Quality control was performed using the Seurat package (v5.1.0) (12), with filtering criteria set to include cells with nFeature_RNA between 200 and 8,000 genes and mt_percent less than 10%, and genes detected in at least 3 cells. DoubletFinder (v2.0.6) was applied at a predicted doublet rate of 7.5% to remove doublets from each sample, ensuring accurate cell type identification and marker gene expression (13). Highly variable genes were identified using the FindVariableFeatures function, and gene expression data were normalized with the LogNormalize method to mitigate experimental noise. To integrate data across different samples and effectively eliminate batch effects, the Harmony algorithm was employed (14). This resulted in an integrated dataset of 189,431 high-quality single-cell transcriptomes. To assess the effectiveness of batch correction, we examined dataset mixing across samples using UMAP visualization and confirmed that cells clustered primarily by biological identity rather than dataset origin. Additionally, principal component distributions before and after Harmony integration were compared to ensure removal of dominant batch-associated variance.

### Cell type annotation and dimensionality reduction

To identify cellular subtypes across RB and control samples, we applied dimensionality reduction and clustering. Principal component analysis (PCA) was used to reduce dimensionality, retaining the top 30PCs for clustering with Seurat’s FindClusters (resolution=0.2). UMAP was used for non-linear projection and visualization. Cell type identification combined with automatic annotation via ScType (15) based on established retinal cell marker genes with manual annotation. The automatic annotation utilized reference data including the ScTypeDB database and curated markers from public datasets. Following automatic labeling, manual refinement was performed by examining marker expression profiles across clusters. The major identified cell types included rod precursor-like cells, cone-like cells, cone precursor-like cells, retinoma-like cells, mature rods, bipolar cells, Müller glia, microglia, as well as two MKI67 positive proliferative cell subpopulations designated MKI67^+^ cells1 and MKI67^+^ cells2.

### Differential gene expression analysis

Differential expression analysis was conducted using the FindMarkers function in Seurat (v5.1.0), applying the Wilcoxon rank-sum test. Comparisons were performed both between the Control and RB groups and across the cell types. Threshold parameters were set as minimum expression proportion (min.pct) greater than 0.2, log2 fold change (logfc.threshold) greater than 0.25, and adjusted p-value below 0.05. Differential gene expression was assessed for each cell type using a “one versus rest” comparison strategy to identify cell type-specific marker genes. For condition-specific comparisons, group-level DEGs were assessed (ident.1 = “RB”, ident.2 = “Control”). Particular attention was paid to DEGs enriched in MKI67^+^ cells2, including the proliferation-associated gene BIRC5.

### Functional enrichment and pathway analysis

Functional enrichment analysis was performed using the RunEnrichment function in SCP (https://github.com/zhanghao-njmu/SCP) for Gene Ontology (GO) biological process (BP) terms. The species parameter was set to “Homo_sapiens”, and differentially expressed genes were filtered with thresholds of “avg_log2FC > 1.0 & p_val_adj < 0.05”. Gene set enrichment analysis (GSEA) was conducted using the fgsea package (v1.32.0)^19^, also based on the GO_BP database with the same gene filtering criteria. Cell cycle analysis employed the CellCycleScoring method implemented in Seurat, utilizing the cc.gene dataset to score cells for S phase and G2/M phase, based on 43 s.genes and 54 g2m.genes, respectively. The top 5 leading-edge genes for the pathway “chromosome segregation” were shown using a GSEA plot.

### Immunofluorescence staining

Immunofluorescence staining was performed on paraffin-embedded retinoblastoma tissue sections using standard deparaffinization, antigen retrieval, blocking, and antibody incubation procedures. Paraffin-embedded retinoblastoma tissue sections were prepared from surgically resected specimens fixed in 10% neutral-buffered formalin for 24 h at room temperature and embedded in paraffin. The paraffin sections of patients with retinoblastoma as pathological results and bake the sections in a 60°C constant temperature oven for 10 minutes, then place the sections in xylene for 10 minutes, repeat soaking in new xylene for 10 minutes, and soak them in 100% anhydrous ethanol, 85% anhydrous ethanol, and 75% anhydrous ethanol for 5 minutes each for dewaxing and hydration. After completion, the tissue slices were placed in a 0.01M citric acid antigen buffer solution (pH 6.0) and gently boiled in a pressure cooker for 15 minutes for antigen repair. After natural cooling to room temperature, the slices were removed and washed three times with PBS for 5 minutes each time. Add 3% BSA dropwise and incubate at room temperature for 30 minutes for sealing. After the sealing is completed, gently wipe off the sealing solution, add the prepared primary antibody (Abclonal, A24431, 1:200) dropwise, slice and place in a wet box, and incubate overnight at 4°C. On the second day, wash the slices three times with PBS on a decolorization shaker for 5 minutes each time. After washing, add the prepared secondary antibody (Thermo, A-31573, 1:200) dropwise and incubate with the secondary antibody at room temperature in the dark for 1 hour. After incubation, wash the slices three times with PBS on a decolorization shaker for 5 minutes each time, and stain the cells with DAPI for 15 minutes. Finally, the film was sealed with an anti-quenching agent, and fluorescent images were taken using a BioTek Bioton Cytation5 instrument.

### Cell lines and culture conditions

The human retinal pigment epithelial cell line (hTERT RPE-1) and the human RB cell lines (Y79 and WERI-Rb-1) were purchased from the American Type Culture Collection (ATCC). The cell lines were cultured in RPMI-1640 (Hyclone, Thermo Scientific) medium supplemented with 10% fetal bovine serum (Hyclone, Thermo Scientific) at 37 °C in an incubator with 5% CO_2_. All of cell lines were confirmed with negative mycoplasma contamination.

### Antibodies

Antibodies against BIRC5 (A24431), GAPDH (A19056), CCND1 (A19038), CHEK2 (A19543) and P21 (A19094) were purchased from Abclonal.

### Plasmid constructs and siRNAs transfection

The full-length human BIRC5 CDS was generated and cloned into the pcDNA3.1 vector. The BIRC5 siRNAs were synthesized by RiboBio. Successfully constructed plasmids or synthesized siRNA were transiently transferred to Y79 cells together with lipo2000, respectively, and then collected after 48–72 hours.

### RNA extraction and real-time quantitative PCR assays

Total RNA from Y79 cells treated with BIRC5-pcDNA3.1 or siRNAs was extracted with a Qiagen RNeasy Mini Kit according to the manufacturer’s instructions. The mRNA expression of the BIRC5 gene was measured using the PerfectStart Green qPCR SuperMix kit. The qPCR results are presented as 2-^ΔΔCt^. Each group is assigned 3 replicates, and the average value is taken.

### Western blot

Collect Y79 cells transfected with BIRC5-siRNA or plasmid, extract total protein, and determine protein concentration using the BCA assay kit. Adjust the protein loading amount to 20 μg/well for loading. The protein samples were electrophoresed on SDS-PAGE, and then wet rotated on the 0.22 μm PVDF membrane. After the membrane transfer is completed, wash the residual transfer solution three times with PBS, and prepare a blocking solution (5% skim milk powder). Place the PVDF membrane in the blocking solution and block it at room temperature for 1 hour. Prepare primary antibodies and incubate overnight at 4°C. On the second day, the membrane was left at room temperature and washed three times with PBS before incubating the secondary antibody at room temperature for 1 hour. After incubation, the protein signals were detected using ECL reagent.

### Cell viability assay

The cell viability was measured using Cell Counting Kit-8 (MedChem Express, Monmouth Junction, NJ, USA) according to the manufacturer’s recommendations. In short, Y79 cells with BIRC5-siRNAs or plasmids were seeded in 96-well plate. Record cell proliferation every 24 hours for 4 days. The number of live cells was evaluated by measuring absorbance at 450nm using a microplate reader (BIO-TEK Instruments, Winooski, VT, USA).

### Transwell assay

Cell migration and invasion assays were performed using Transwell chambers (8.0 μm pore size). Cells (3 × 10^4^) were seeded in the upper chamber, and medium containing 10% FBS was added to the lower chamber. After 24 h incubation, cells were fixed, stained with crystal violet, and quantified under a microscope. Transwell chamber (coated or uncoated with matrix gel, pore size 8.0 μm) placement: Add 500 μL of culture medium containing 10% FBS to the lower chamber (24 well plate), place the Transwell chamber in the 24 well plate, take 200 μL of cell suspension and add it to the upper chamber, with a cell count of 30000/well. Incubate in the incubator for 24 hours. After 24 hours of cultivation, discard the culture medium in the chamber and wash twice with PBS for 3 minutes each time. Place the small chamber into a 24-well plate, add 600 μL of paraformaldehyde, and fix at room temperature for 30 minutes. After fixation is completed, remove the small chamber, discard the residual fixative in the chamber, and wash twice with PBS for 3 minutes each time. Transfer the chamber to a well pre-filled with 0.1% crystal violet solution, allowing the staining solution to completely immerse the membrane, and stain at room temperature for 15–30 minutes. Wash twice with ddH_2_O for 3 minutes each time to remove residual crystal violet. After the experiment, gently wipe the upper side of the chamber with a cotton swab to remove non-specific staining. After natural drying, take photos and count under a microscope. stained cells were counted in at least five randomly selected, non-overlapping fields per membrane under a light microscope at 200× magnification. Images were captured using identical exposure settings across all groups. Cell counts were quantified using ImageJ software (NIH, version 1.53). All analyses were performed in triplicate independent experiments, and quantification was conducted by investigators blinded to group allocation to minimize bias.

### Statistical analysis

All statistical analyses were performed using GraphPad Prism (version 10.0). Data are presented as mean ± standard deviation (SD) unless otherwise specified. Prior to statistical testing, data normality was assessed using the Shapiro–Wilk test, and homogeneity of variances was evaluated using Levene’s test. For comparisons between two groups, unpaired two-tailed Student’s t-test was used when data followed a normal distribution with equal variances. When these assumptions were not met, the non-parametric Mann–Whitney U test was applied. All experiments were performed with at least three independent biological replicates (n ≥ 3). For assays involving technical repeats, the mean of technical replicates was used for statistical analysis. A P value < 0.05 was considered statistically significant.

## Results

### Distinctive cell types in retinoblastoma samples

**Figure 1:**
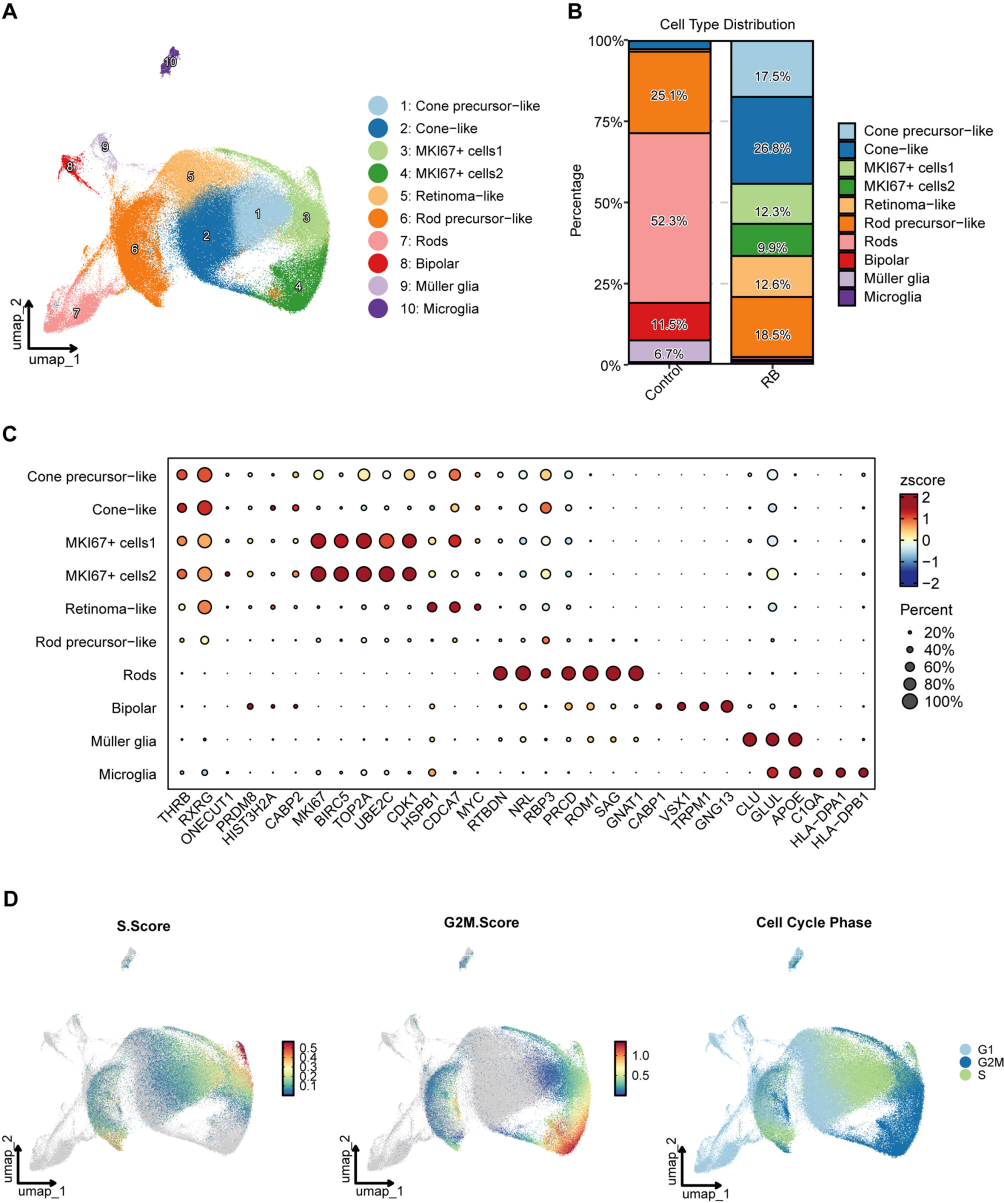
Single-cell transcriptomic landscape and identification of proliferative tumor subpopulations in retinoblastoma. **(A)** UMAP visualization of 189,431 single cells from retinoblastoma and control retinal tissues, colored by cell type annotation. Ten major cell populations are identified, including cone precursor-like, cone-like, MKI67^+^ cells1, MKI67^+^ cells2, retinoma-like, rod precursor-like, rods, bipolar cells, Müller glia, and microglia; **(B)** proportional distribution of cell types in control versus retinoblastoma samples. Stacked bar plots show enrichment of proliferative and cone-lineage populations in RB and relative depletion of differentiated retinal cell types; **(C)** dot plot showing expression of representative marker genes across cell types. Dot size represents the percentage of cells expressing each gene, and color intensity indicates scaled expression (z-score). Proliferative clusters (MKI67^+^ cells1/2) show strong enrichment of cell cycle–associated genes; **(D)** cell cycle state analysis across all cells. Left: S-phase score; middle: G2/M-phase score; right: assigned cell cycle phase. MKI67^+^ cells2 are enriched in G2/M phase, indicating high proliferative activity.

The integrated single-cell RNA sequencing dataset comprised 189,431 cells from 13 retinoblastoma tumors and 3 normal fetal retina samples. Unsupervised clustering identified 10 distinct cell populations, visualized using UMAP (**Figure 1A**), including cone precursor-like, cone-like, retinoma-like, rod precursor-like, rods, bipolar cells, Müller glia, microglia, and two MKI67-positive proliferative subpopulations. As shown in the proportional distribution analysis (**Figure 1B**), cone precursor-like (17.5%), cone-like (26.8%), retinoma-like (12.6%), and MKI67^+^ subpopulations (cells1: 12.3%; cells2: 9.9%) were enriched in retinoblastoma samples, whereas differentiated retinal cell types such as rods, bipolar cells, Müller glia, and microglia were more abundant in control samples, indicating tumor-associated expansion of proliferative and lineage-specific compartments. Marker gene expression profiles across clusters (**Figure 1C**) confirmed cell identity. Cone lineage populations expressed canonical markers including *RXRG*, *THRB*, *GNGT2*, and *GNAT2*, while rod-associated clusters expressed *NRL*, *RBP3*, and *PRCD*. Notably, MKI67^+^ cells exhibited strong enrichment of proliferation-associated genes such as *MKI67*, *TOP2A*, *BIRC5*, *UBE2C*, and *CDK1*, consistent with an actively cycling tumor population. Cell cycle scoring further demonstrated that the MKI67^+^ cells2 subpopulation was predominantly enriched in G2/M-phase signatures (**Figure 1D**), indicating heightened mitotic activity compared to other cell types. In contrast, non-proliferative retinal populations were primarily distributed in G1 phase. These findings collectively identify MKI67^+^ cells2 as the most proliferative and potentially aggressive tumor compartment in retinoblastoma.

### MKI67^+^ cells enriched in BIRC5 cell proliferation pathways

**Figure 2:**
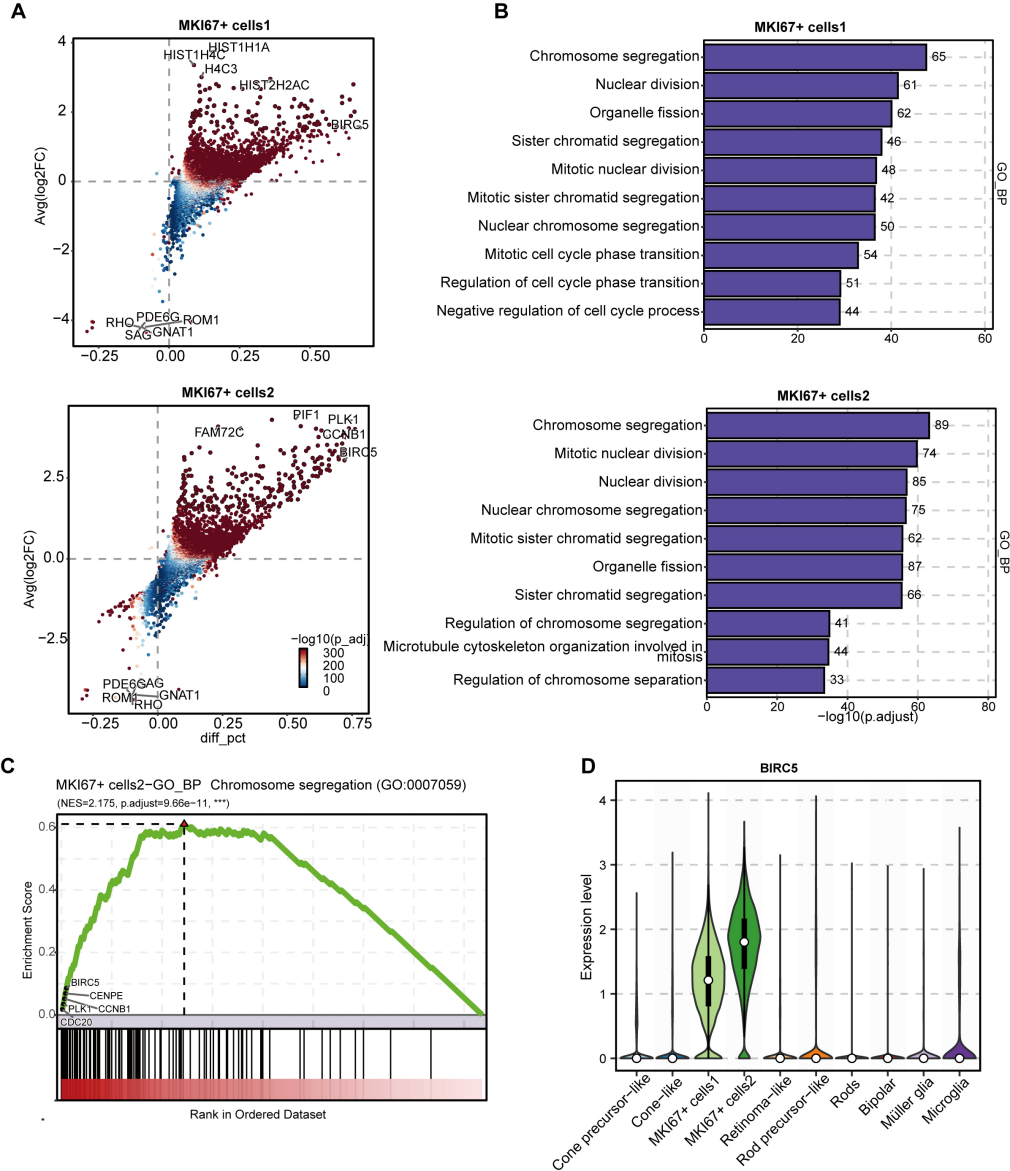
Differential expression, enriched pathways, and cell cycle marker analysis in MKI67^+^ subpopulations. **(A)** Differential gene expression analysis for MKI67-positive cell subpopulations. Scatter plots display differentially expressed genes between subpopulations of MKI67^+^ cells1 (top) and cells2 (bottom) in RB single-cell transcriptomes. Each point represents a gene, with color indicating –log10(adjusted P-value), and y-x axes showing average log2 fold-change versus percentage difference (diff_pct). The top genes were labeled.; **(B)** GO enrichment analysis of biological processes in MKI67^+^ subpopulations. Horizontal bar plots show significantly enriched GO terms for MKI67^+^ cells1 (top) and MKI67^+^ cells2 (bottom), with bars representing –log10 (adjusted P-value), and numbers indicating the count of genes enriched for each process. The most enriched categories relate to chromosome segregation, mitotic division, and cell cycle phase transitions; **(C)** GSEA for the biological process “chromosome segregation” in MKI67^+^ cells2. The enrichment score curve visualizes the overrepresentation of genes involved in chromosome segregation across the ranked gene list, with core enrichment genes such as BIRC5, CENPF, PLK1, CCNB1, and CDCA2 highlighted; **(D)** Violin plot of BIRC5 gene expression across major retinal cell types. Violin plots depict the distribution of BIRC5 expression levels in each cell type, indicating the highest enrichment in MKI67^+^ cells2.

Differential gene expression analysis highlighted that both MKI67^+^ cells1 and MKI67^+^ cells2 exhibit more active gene expression compared to other cell types, though they differ in specific gene profiles. MKI67^+^ cells1 are highly enriched in histone genes such as HIST1H1A, HIST1H4C, and HIST2H2AC, whereas MKI67^+^ cells2 show heightened expression of cell cycle genes including PLK1 and CCNB1 (**Figure 2A**). Functional enrichment analysis revealed these subpopulations are strongly enriched in crucial biological processes related to the cell cycle and mitosis, including chromosome segregation and mitotic nuclear division (**Figure 2B**). Among the top genes within the chromosome segregation pathway are CCNB1 and PLK1 (G2/M transition regulators), CENPE and BIRC5 (spindle checkpoint genes), and CDC20 (cytokinesis gene) (**Figure 2C**). The coordinated action of these genes facilitates rapid and efficient cell division. Notably, BIRC5 is highly expressed in 79.9% of MKI67^+^ cells1 (mean expression 1.166 ± 0.587) and 93.7% of MKI67^+^ cells2 (mean 1.733 ± 0.643), while only minimally expressed (<0.16) in other cell types (**Figure 2D**). The marked elevation of BIRC5 in the MKI67^+^ cells2 subpopulation correlates closely with their pronounced malignant characteristics, suggesting BIRC5 as a critical driver in retinoblastoma progression. Therefore, BIRC5 emerges as a promising molecular target for therapeutics aimed at highly proliferative RB cells and warrants further molecular biological investigation. Notably, BIRC5 was co-expressed with other mitotic regulators including AURKB, TPX2, and KIF2C, forming a coordinated transcriptional module associated with chromosome segregation and spindle assembly. This four-gene module was highly enriched in the MKI67^+^cells2 population, indicating that dysregulated mitotic machinery characterizes the most proliferative RB tumor compartment. In addition to BIRC5, several mitotic regulators including AURKB, TPX2, and KIF2C were also highly ex-pressed within the MKI67^+^cells2 cluster. These genes function cooperatively in mitotic regulation AURKB and BIRC5 form components of the chromosomal passenger complex, TPX2 regulates Aurora kinase–dependent spindle assembly, and KIF2C controls microtubule dynamics during chromosome segregation. Their coordinated upregulation therefore suggests the presence of a coherent mitotic regulatory program driving the highly proliferative RB tumor state.

### BIRC5 was significantly overexpressed in retinoblastoma patients and cells

**Figure 3:**
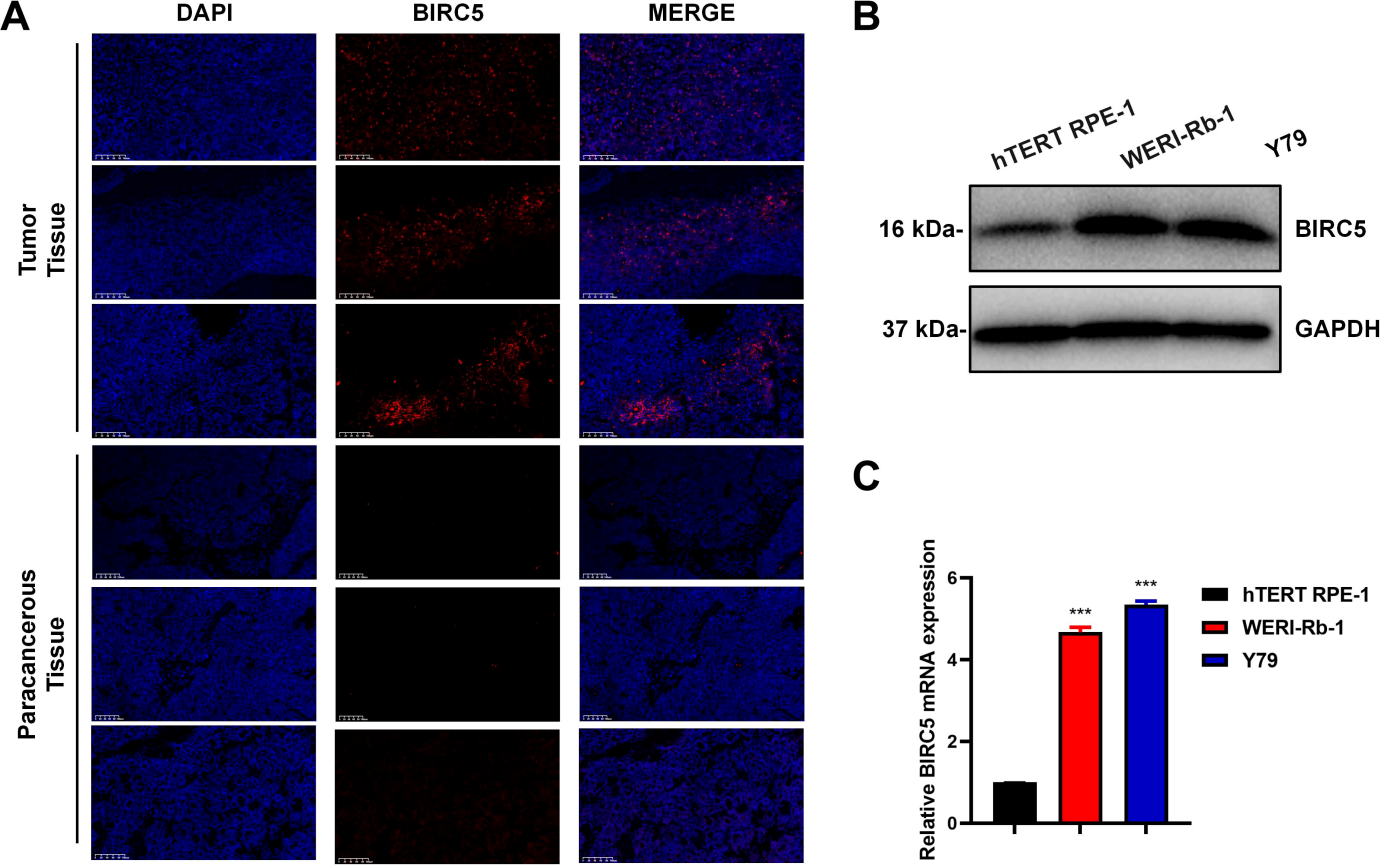
BIRC5 was significantly overexpressed in retinoblastoma patients and cells. **(A)** Immunofluorescence staining was used to examine the expression level of BIRC5 in three retinoblastoma patient tissues and paired adjacent paracancerous tissues. Scale bar, 100 μm. **(B, C)** the mRNA and protein levels of BIRC5 gene were determined in hTERT RPE-1, Y79 and WERI-Rb-1 cells by RT-qPCR **(B)** and Western blot **(C)** (n=3, ***P < 0.001).

To investigate the expression of BIRC5 in the progression of retinoblastoma, we first collected tissue sections from three patients with retinoblastoma for tissue fluorescence staining. The results indicated that BIRC5 was significantly overexpressed in retinoblastoma tissues compared to paracancerous tissues (**Figure 3A**). We evaluated BIRC5 expression in human retinal pigment epithelial cells (hTERT RPE-1) and retinoblastoma cell lines (WERI-Rb-1 and Y79). Western blot analysis demonstrated markedly increased BIRC5 protein expression in WERI-Rb-1 and Y79 cells compared with hTERT RPE-1 cells (**Figure 3B**). Consistently, RT-qPCR analysis showed significantly higher BIRC5 mRNA expression levels in retinoblastoma cell lines relative to normal retinal epithelial cells (**Figure 3C**). Collectively, these findings demonstrate that BIRC5 is aberrantly upregulated in retinoblastoma tissues and tumor cell lines.

### Overexpression of BIRC5 could significantly promote the progression of retinoblastoma cells

**Figure 4:**
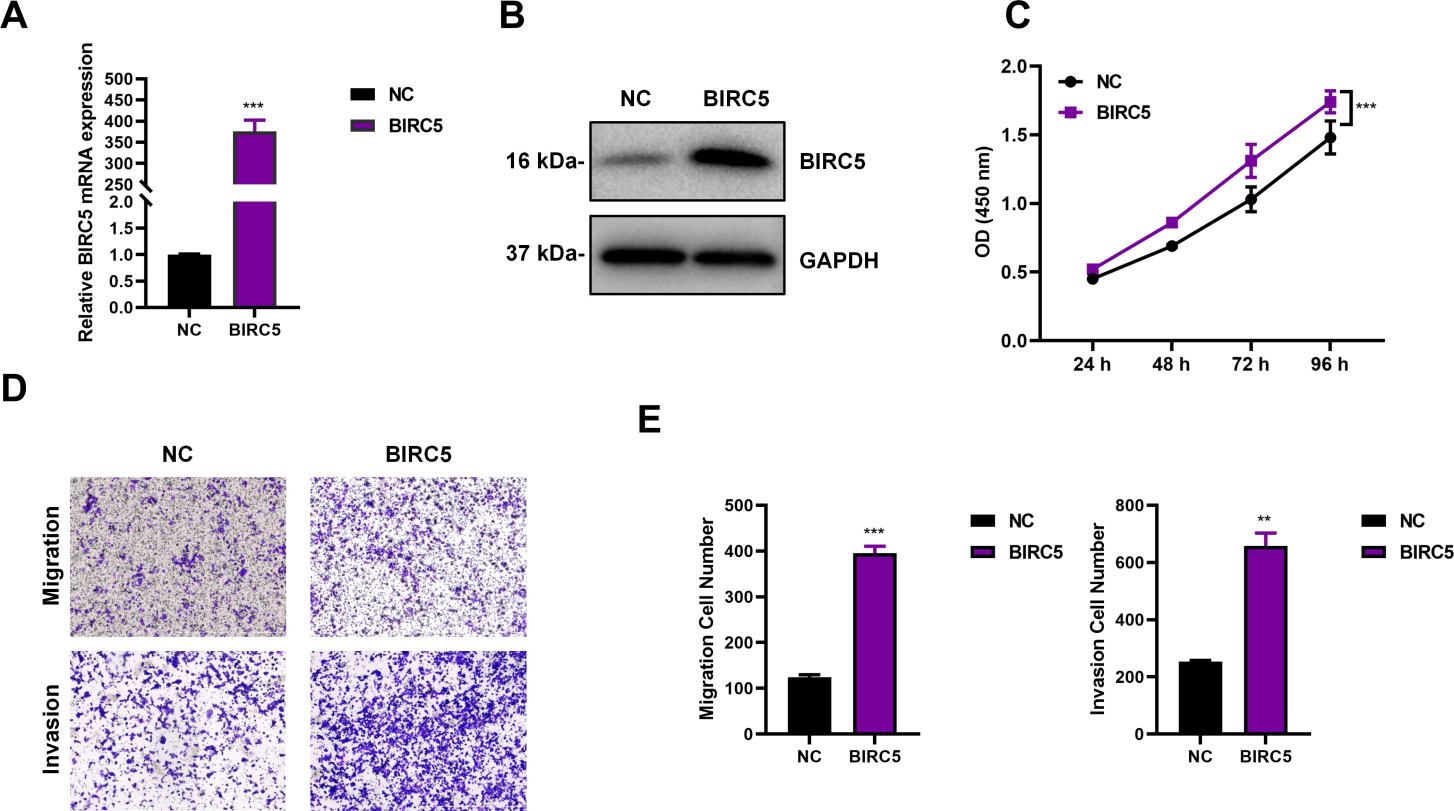
Overexpression of BIRC5 could significantly promote the progression of retinoblastoma cells. **(A)** RT-qPCR analysis showing increased BIRC5 mRNA expression following BIRC5 overexpression. **(B)** Western blot analysis confirming elevated BIRC5 protein expression in BIRC5-overexpressing Y79 cells. GAPDH served as the loading control. **(C)** CCK-8 assays showing enhanced proliferative ability of Y79 cells following BIRC5 overexpression. **(D)** Representative images of Transwell migration and invasion assays in control and BIRC5-overexpressing Y79 cells. **(E)** Quantitative analysis of migrated and invaded cells shown in panel **(D)**. Data are presented as mean ± SD [n = 3 for **(A, B, D, E)** n = 5 for **(C)**]. **P < 0.01, ***P < 0.001.

To explore the role of BIRC5 in the progression of retinoblastoma, we constructed a BIRC5 overexpression plasmid and transfected it into the Y79 cell line. RT-qPCR and Western blot results indicated successful overexpression of BIRC5, with a significant increase in its expression level (**Figures 4A, B**). The CCK8 assay demonstrated that overexpression of BIRC5 significantly promoted the proliferative ability of Y79 cells (**Figure 4C**). The Transwell assay showed that overexpression of BIRC5 significantly enhanced the invasive and migratory abilities of Y79 cells (**Figures 4D, E**). The above data suggested that overexpression of BIRC5 could significantly promote the malignant progression of retinoblastoma cells.

### Knockdown of BIRC5 could significantly inhibit the progression of retinoblastoma cells

**Figure 5:**
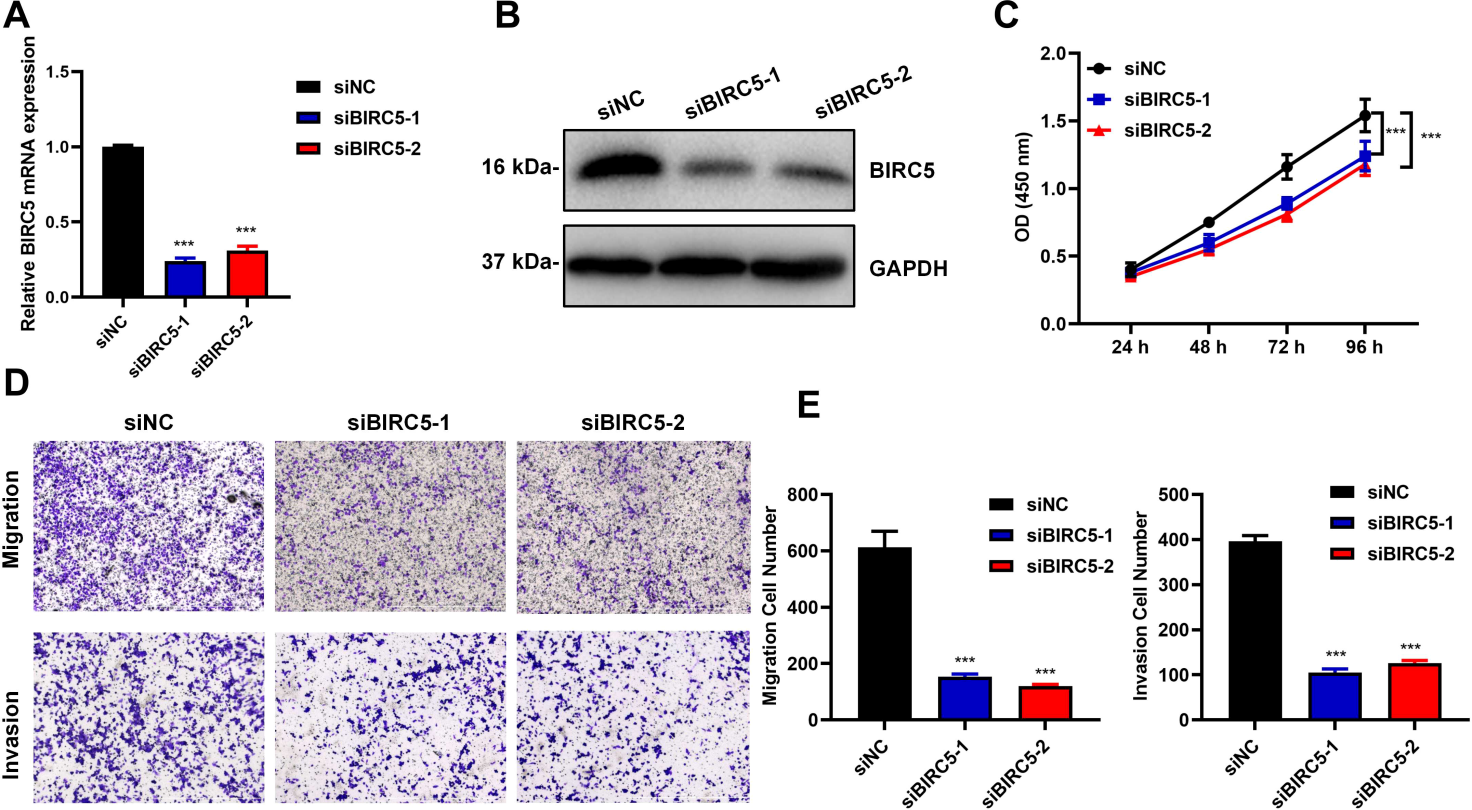
Knock down of BIRC5 could significantly inhibit the progression of retinoblastoma cells. SiRNA-NC and BIRC5-siRNA was transiently transfected into Y79 cell respectively. **(A)** RT-qPCR showed decreased mRNA expression of BIRC5 in BIRC5-suppressed Y79 cell. (n=3, **P < 0.01, ***P < 0.001). **(B)** Western blot showed decreased protein expression of BIRC5 in BIRC5-suppressed Y79 cell. **(C)** CCK8 assay was used to detect the ability of cell proliferation. (n=5, ***P < 0.001). **(D)** Transwell experiment was used to measure the ability of cell migration and invasion in BIRC5-suppressed Y79 cell. Scale bar, 1000 μm. **(E)** The quantifiable data of **(D)**. (n=3, ***P < 0.001).

We synthesized siRNA targeting the BIRC5 gene and transfected it into the Y79 cell line. RT-qPCR and Western blot results indicated a significant decrease in both RNA and protein levels following the transfection of BIRC5 siRNA (**Figures 5A, B**). CCK8 assay revealed that knockdown of BIRC5 significantly inhibited the proliferation of Y79 cells (**Figure 5C**). The transwell assay demonstrated that knockdown of BIRC5 notably suppressed the invasion and migration abilities of Y79 cells (**Figures 5D, E**). These findings suggested that knockdown of BIRC5 could significantly hinder the malignant progression of retinoblastoma cells.

### BIRC5 promoted the progression of retinoblastoma via accelerating the cell cycle process

**Figure 6:**
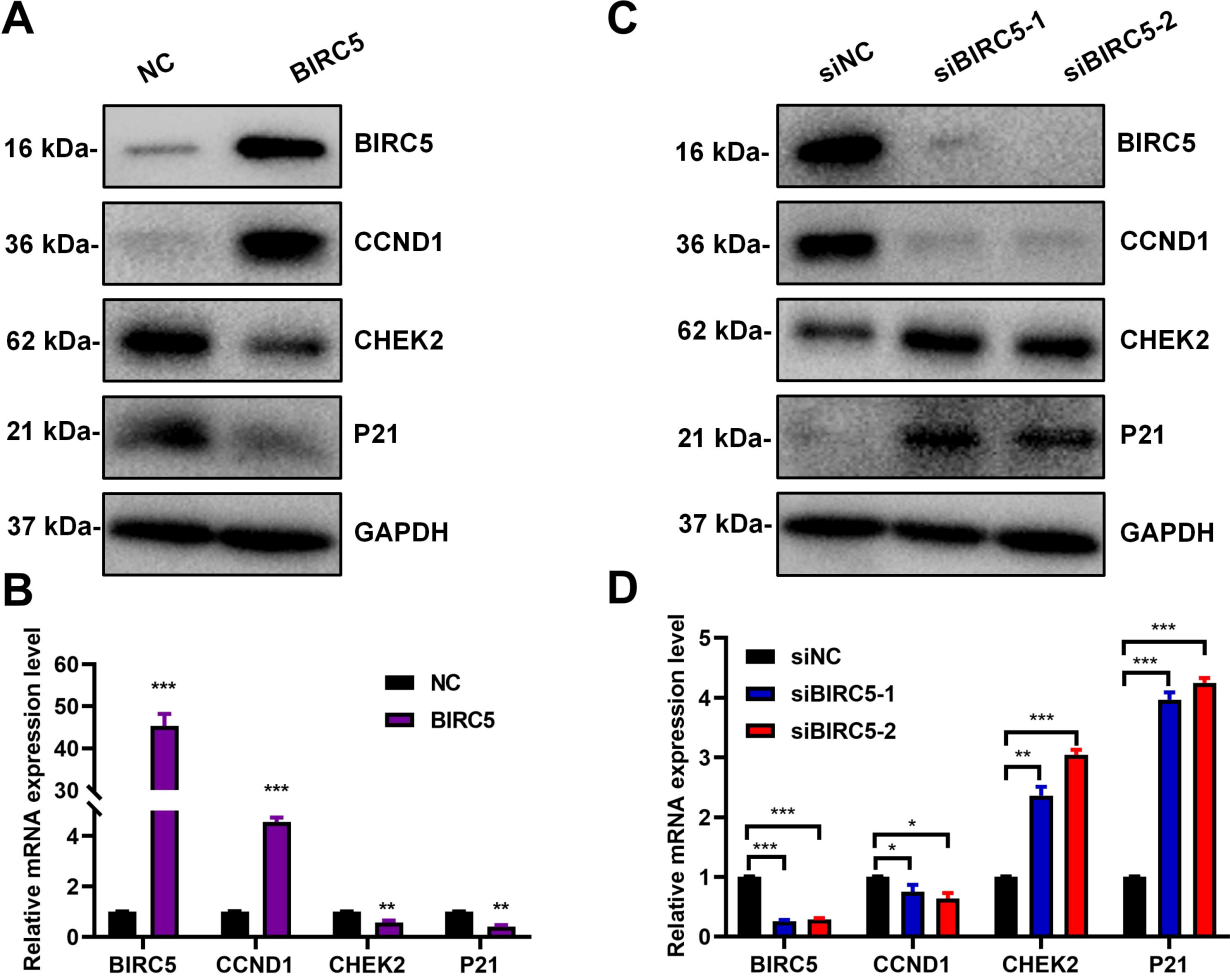
BIRC5 promoted the progression of retinoblastoma via accelerating the cell cycle process. **(A, B)** Flag-NC and Flag-BIRC5 was transiently transfected into Y79 cell respectively, and the mRNA and protein levels of cell cycle relative genes were measured by Western blot **(A)** and RT-qPCR **(B)**. (n=3, **P < 0.01, ***P < 0.001). **(C, D)** SiRNA-NC and BIRC5-siRNA was transiently transfected into Y79 cell respectively, and the mRNA and protein levels of cell cycle relative genes were measured by Western blot **(C)** and RT-qPCR **(D)**. (n=3, *P < 0.05, **P < 0.01, ***P < 0.001).

Previous functional enrichment analysis revealed MKI67^+^ cells2 subpopulations which highly expressed BIRC5 was strongly enriched in crucial biological processes related to the cell cycle (**Figure 2B**), suggesting that BIRC5 may promote the malignant progression of retinoblastoma by accelerating the cell cycle progression. We screened key regulators involved in the cell cycle progression and detected their expression levels through Western blot and RT-qPCR. The results indicated that overexpression of BIRC5 significantly promoted the expression of CCND1 and significantly inhibited the expression of checkpoint kinase CHEK2 and its downstream gene P21 (**Figures 6A, B**). Conversely, knockdown of BIRC5 significantly inhibited the expression of CCND1 and significantly promoted the expression of checkpoint kinase CHEK2 and its downstream gene P21 (**Figures 6C, D**). These findings implied that BIRC5 may promote the progression of retinoblastoma cells by accelerating the cell cycle progression.

## Discussion

The integrated dataset included 13 independent RB tumors, which improves statistical robustness compared with prior single-cell studies of retinoblastoma, integrating 189,431 single cells from tumors and normal fetal retina to uncover lineage-restricted regulators of malignant proliferation. Through this analysis, we identify a distinct subpopulation of MKI67 high cells, specifically MKI67^+^cells2 that exhibit transcriptional features of cell cycle dysregulation, chromosomal instability, and mitotic checkpoint failure. Among this compartment, BIRC5 (survivin) emerged as the most selectively and highly upregulated gene, with functional validation demonstrating its essential role in promoting RB cell proliferation, invasion, and resistance to checkpoint control. Our data collectively position BIRC5 as a nodal driver of the malignant apex in RB tumors, acting through activation of the CCND1/CDK4 axis and suppression of the CHEK2–p21 pathway to bypass DNA damage checkpoints. These findings refine our understanding of intratumoral heterogeneity in RB and identify a tractable, mechanistically validated therapeutic target within its most aggressive cellular subpopulation.

Single-cell transcriptomics has revolutionized our understanding of pediatric solid tumors by resolving lineage hierarchies and revealing cell-type–specific transcriptional programs otherwise obscured in bulk tissue analyses. In RB, traditionally viewed as a genetically simple tumor driven solely by biallelic inactivation of RB1 (16), recent work suggests considerable transcriptional complexity and variability in differentiation states (17). In our study, unsupervised clustering revealed cone precursor-like and cone-like cells as dominant cell states within RB tumors, corroborating the hypothesis that RB arises from maturing cone precursors. Notably, we extend these insights by identifying a transcriptionally unique, highly proliferative MKI67^+^cells2 subpopulation absent in normal fetal retina characterized by overexpression of mitotic checkpoint and chromosomal instability genes, particularly BIRC5, PLK1, and AURKB (18). Consistent with recent comprehensive analyses of retinoblastoma biology and therapeutic strategies (19), our findings support the concept that tumor progression is driven by distinct proliferative subpopulations characterized by dysregulated cell cycle and checkpoint pathways.

BIRC5, located on chromosome 17q25, encodes survivin, a bifunctional protein belonging to the inhibitor of apoptosis protein (IAP) family and the chromosomal passenger complex (CPC), which coordinates mitotic spindle formation, chromosome alignment, and cytokinesis (20). In MKI67^+^ cells2, BIRC5 was expressed in 93.7% of cells with a mean expression level of 1.733 ± 0.643 (normalized log counts), compared to <0.16 in all other clusters. Its expression was negligible in cone precursors from normal fetal retina (E17–E22), as confirmed via integrated analysis with publicly available datasets (21). Our findings parallel reports of survivin overexpression in other embryonal tumors such as neuroblastoma (22), medulloblastoma (23), and high-grade glioma (24), yet its role in RB has remained poorly characterized until now. Importantly, BIRC5 expression was accompanied by upregulation of AURKB, TPX2, and KIF2C, indicating that survivin operates within a broader mitotic regulatory network rather than as an isolated factor. These genes collectively regulate chromosomal passenger complex activity, spindle assembly, and microtubule dynamics, suggesting that dysregulated mitotic machinery underlies the proliferative RB subpopulation identified in this study.

Functional validation studies in RB cell lines (Y79, WERI-Rb1) confirmed BIRC5’s mechanistic role in promoting malignant features. BIRC5 overexpression increased EdU incorporation by 48.2% (*p* < 0.01) and enhanced colony formation (2.3-fold increase, *p* < 0.05) and Matrigel invasion (2.1-fold, *p* < 0.05). Conversely, BIRC5 knockdown via siRNA induced significant G1 arrest (increase in G1-phase cells from 48.1% to 69.4%, *p* < 0.01) and activated apoptosis (Annexin V+ cells increased by 3.6-fold). These effects were accompanied by downregulation of CCND1 and CDK4 and upregulation of the DNA damage response proteins CHEK2 and CDKN1A (p21), consistent with previous findings in colorectal and breast cancer models (25). These data suggest that BIRC5 functions as a molecular rheostat balancing proliferative drive and checkpoint evasion. These data suggest that BIRC5 functions as a molecular rheostat balancing proliferative drive and checkpoint evasion. Importantly, BIRC5 did not act in isolation but was co-expressed with other mitotic regulators including AURKB, TPX2, and KIF2C, suggesting the presence of a coordinated mitotic regulatory program driving the malignant proliferative state identified in RB.

Our data suggest that BIRC5 is functionally associated with modulation of the CCND1/CDK4 axis and repression of the CHEK2–p21 tumor suppressor pathway; however, these findings are based on expression and perturbation analyses and do not establish direct causal or physical interactions. CHEK2 is a DNA damage transducer kinase that activates p21 to enforce G1/S arrest. BIRC5 overexpression likely dampens CHEK2 signaling, thereby permitting continued proliferation despite genotoxic stress. This feature is shared with several aggressive pediatric cancers such as rhabdomyosarcoma and malignant rhabdoid tumors, where G1 checkpoint bypass and DNA repair suppression contribute to therapy resistance (26, 27). Further supporting its role in checkpoint suppression, survivin-deficient cells accumulate DNA damage and undergo p53-mediated apoptosis (28), and BIRC5 overexpression facilitates oncogenic transformation by neutralizing CHK1/2 (29). These findings therefore define a functional association between BIRC5 and cell cycle checkpoint regulation in RB, while highlighting the need for further mechanistic validation to establish direct causal links.

Importantly, BIRC5 expression is highly selective for malignant RB tissue. Immunofluorescence staining in FFPE tumor sections from 12 patients revealed nuclear survivin expression in 78.4 ± 9.3% of tumor cells but negligible staining in adjacent retina or fetal control retina (n=3). This tumor-specific expression pattern supports BIRC5 as an ideal therapeutic target. Several small-molecule inhibitors of survivin have been explored, including YM155, which suppresses BIRC5 transcription (30) and shepherdin, which disrupts its interaction with heat shock proteins. Although no survivin-targeted agents are FDA-approved, clinical trials are ongoing for YM155 (NCT02012192) and survivin peptide-based vaccines (EMD640744, NCT01193484). Beyond therapeutic implications, our study provides insight into the potential developmental trajectory of retinoblastoma. Cone precursor-like cells expressing RXRG, THRB, and CRX are strongly associated with tumor cell populations and may represent a plausible cell-of-origin, consistent with prior studies. In this context, MKI67^+^ cells1 and MKI67^+^ cells2 may reflect distinct proliferative states along a continuum of malignant progression rather than strictly defined sequential stages. MKI67^+^ cells1 exhibited elevated expression of histone genes (HIST1H2AC, HIST1H4C) with relatively lower activation of mitotic regulators, suggesting a less advanced proliferative state. In contrast, MKI67^+^ cells2 demonstrated enrichment of G2/M checkpoint dysregulation and genomic instability pathways, consistent with a more aggressive proliferative phenotype. These observations are consistent with previously proposed models of retinoblastoma development, in which cone lineage–associated cells undergo progressive transcriptional and cell cycle reprogramming during tumor evolution (31), where epigenetically altered cone precursors progress toward malignancy via cell cycle reprogramming and chromatin remodeling. From a therapeutic perspective, co-targeting BIRC5 and CDK4/6 may suppress the most aggressive proliferative subpopulations. CDK4/6 inhibitors (palbociclib) have shown promise in pediatric sarcomas (32), and RB cell lines exhibit partial sensitivity *in vitro*. Given survivin’s role in chemoresistance, dual inhibition could enhance response to conventional chemotherapeutics such as topotecan and carboplatin. Moreover, the highly restricted expression of BIRC5 in RB opens the door for immunotherapeutic strategies. Recent advances in T-cell receptor–engineered therapies (TCR-T) targeting survivin peptides (e.g., TBI-1301) underscore the feasibility of this approach, especially in HLA-A*02:01-positive patients (33). While BIRC5 (survivin) is a well-established regulator of mitosis and apoptosis in multiple cancer types, including retinoblastoma, our findings do not aim to redefine its canonical molecular function. Instead, this study provides a refined, cell-state–resolved perspective by demonstrating that BIRC5 is selectively enriched within a transcriptionally distinct MKI67^+^ cells2 subpopulation, representing the most proliferative and genomically unstable compartment of the tumor. This highlights BIRC5 as a lineage-restricted dependency rather than a universally acting oncogenic driver in RB.

Several important considerations shape the interpretation and future extension of our findings. Although the integrated scRNA-seq atlas includes 189,431 high-quality cells from four independent datasets, rare or transient subpopulations particularly those involved in early tumorigenesis or treatment resistance may still be underrepresented. Expanded sampling, especially from treatment-naïve and relapsed RB cases, could offer a more comprehensive cellular landscape. Additionally, while the Y79 and WERI-Rb-1 cell lines provide valuable models for functional validation, they may not fully capture the intratumoral heterogeneity or microenvironmental context observed *in vivo*. The application of more physiologically relevant models, such as patient-derived organoids or xenografts, will be essential to refine our understanding of BIRC5-mediated tumor dynamics and therapeutic responses.

Moreover, while our study highlights BIRC5 as a key regulator of retinoblastoma proliferation, it functions within the chromosomal passenger complex (CPC) alongside proteins such as INCENP and AURKB. Our single-cell analysis further revealed coordinated upregulation of BIRC5 with AURKB, TPX2, and KIF2C, suggesting the presence of a mitotic regulatory module associated with spindle assembly and chromosome segregation in highly proliferative tumor cells. Although functional validation in this study focused on BIRC5 due to its strongest differential expression and therapeutic relevance, this four-gene module may provide a more comprehensive marker of aggressive proliferative states. Future studies incorporating larger patient cohorts and integrative multi-omics approaches will be required to determine whether module-based signatures improve tumor stratification and prognostic prediction compared with single-gene markers.

Several limitations should be considered. Although Harmony-based integration reduced dataset-specific variation, residual batch effects related to sequencing depth and sample heterogeneity cannot be fully excluded; however, the consistent identification of the MKI67^+^ cells2 subpopulation and its associated BIRC5 enrichment across independent datasets supports the robustness of our findings. In addition, functional experiments were primarily conducted in Y79 cells, and the lack of *in vivo* validation limits direct translational interpretation. Future studies using patient-derived organoids and xenograft models will be essential to validate these observations in more physiologically relevant systems. Finally, the mechanistic conclusions presented here are based on correlative pathway changes following BIRC5 perturbation. Although consistent alterations in CCND1, CDK4, CHEK2, and p21 suggest involvement in G1/S checkpoint regulation, direct causal relationships were not established. Further investigation using rescue experiments, pharmacological pathway inhibition (e.g., CDK4/6 inhibitors such as palbociclib), and protein–protein interaction analyses will be necessary to define the precise signaling hierarchy and confirm pathway dependency.

## Conclusion

This study delineates the transcriptional and functional architecture of malignant cell states in retinoblastoma by integrating single-cell transcriptomic profiling with mechanistic validation. We identify a distinct MKI67^+^ cells2 subpopulation characterized by cell cycle hyperactivation and chromosomal instability, in which BIRC5 (survivin) is selectively and highly expressed. Functional assays confirm that BIRC5 is not merely a marker but an essential regulator of RB cell proliferation and invasion, acting through the CCND1–CDK4 axis and suppression of CHEK2–p21–mediated checkpoint control. BIRC5 is virtually absent in normal retina and enriched specifically in the most proliferative tumor compartments, highlighting its potential as a lineage-restricted therapeutic target. By resolving the proliferative hierarchy within RB and pinpointing a mechanistically actionable driver, this work provides a framework for targeting the malignant apex of RB tumors and informs broader strategies for treating pediatric cancers driven by dysregulated cell cycle networks.

## Data Availability

The original contributions presented in the study are included in the article/Supplementary Material. Further inquiries can be directed to the corresponding authors.
